# Application of *Zataria multiflora *Boiss. and *Cinnamon zeylanicum *essential oils as two natural preservatives in cake

**Published:** 2013

**Authors:** Habibe Kordsardouei, Mohsen Barzegar, Mohamad Ali Sahari

**Affiliations:** 1 *Department of Food Science and Technology, Tarbiat Modares University, Tehran, I. R. Iran*

**Keywords:** *Cinnamon zeylanicum*, Functional Food, Natural Preservative, Sponge Cake, * Zataria multiflora *Boiss

## Abstract

**Objective:** Oxidation of oils has an important effect on nutritional and organoleptic properties of foodstuffs. Nowadays, new tendency has created a necessity to use natural compounds such as essential oils for producing functional foods. In this study, antioxidant, antifungal, and organoleptic properties of *Zataria multiflora *Boiss. (ZMEO) and *Cinnamon zeylanicum* essential oils (CZEO) have been checked as two natural preservatives in the cakes.

**Materials and Methods**: The antioxidant activity of essential oils were determined by measuring thiobarbituric, peroxide, and free fatty acid values of prepared cakes during 60 days storage at 25 ˚C. Antifungal properties of essential oils were determined and given as the ratio of colony number in samples containing ZMEO and CZEO to the control.

**Results**: Different concentrations of essential oils prevented oxidation rate and reducd preliminary and secondary oxidation products compared with butylate hydroxyanisole (BHA (100 and 200 ppm)) and control cakes. Moreover, ZMEO and CZEO at three concentrations (500, 1000, and 1500 ppm) reduced the fungal growth more than samples containing BHA (100 and 200 ppm) and the control.

**Conclusion**: Our results showed that optimum concenteration of ZMEO and CZEO for using in the cakes was 500 ppm therefore it can be replaced instead of synthetic preservatives in foodstuffs.

## Introduction

Foods containing fat and oils, oxidize slowly during storage. Diferent oxidation products cause rancidity and reduction of the sensory properties of the food products (Reddy et al., 2005 ▶). Cake manufacturers face some problems such as lipid oxidation and fungal growth which reduce the shelf-life of their products (Lean and Mohamed, 1999 ▶). Using antioxidants and preservatives can solve these problems. Synthetic additives such as butylated hydroxyl anisole (BHA) and butylated hydroxy toluene (BHT) have been used as antioxidants in foodstuffs. 

The use of these synthetic antioxidants has been restricted because of their toxicity (Reddy et al., 2005 ▶). Recently, natural plants have attended as a source of biologically active substances such as antioxidant, antifungal, antimutagen, and anticarcinogen. Aromatic plants, spices, and fruit powders have been used in foodstuffs (Bajaj and Urooj, 2006 ▶). Badei et al. (2000) ▶ used anise and cumin oils as natural antioxidants for stabilisation of bakery products. Reddy et al. (2005) ▶ evaluated antioxidant activity of some plant extracts and their application in biscuits. Fazel et al. (2009) ▶ used tea and sesame seed oils as natural antiradical in fish oil system. 

Most researches on antimicrobial activity of plant extracts have attended to the common food-borne pathogens and fungi such as *Escherichia coli*, *Staphylococcus aureus*, *Klebsiella pneumonie*, *Listeria monocytogenes*, *Campylobacter jejuni*, and also *Candida* spp., *Zygosaccharomyses* spp., *Fusarium* spp., *Rhizopus* spp., and *Penicillium* spp. (Ahmadi et al., 2010 ▶). Omidbeygi et al. (2007) evaluated antifungal activity of thyme, summer savory, and clove essential oils against *Aspergillus flavus *in liquid medium and tomato paste. Antioxidant, antimicrobial, and antimutagenic activities of pistachio green hull extract were evaluated by Rajaei et al. (2010) ▶. Recently, researchers have focused on medicinal plants for extracting natural antioxidants that can replace synthetic kinds that might be carcinogenic (Whysner et al., 1994 ▶) and even toxic (Moure et al., 2001 ▶). Furthermore, a lot of plants used as spices have also antimicrobial properties that inhibit the growth of food-borne pathogens (Ahmadi et al., 2010 ▶).

*Zataria multiflora* Boiss. is a member of Laminaceae family that geographically grows in Iran, Pakistan, and Afghanistan (Ali et al., 2000 ▶; Hosseinzadeh et al., 2000 ▶). This plant with folk name of Avishan Shirazi (in Iran) has been used as anesthetic, antiseptic, and antispasmodic (Hosseinzadeh et al., 2000 ▶; Jafari et al., 2011 ▶). It is widely used as flavor composition in extensive range of foodstuffs in Iran. The main constituents of ZMEO are phenolic compounds such as carvacrol and thymol (Shaffiee and Javidnia, 1997 ▶). Moosavy et al. (2008) ▶ evaluated the effect of *Z. multiflora* Boiss. essential oil and nisin on *Salmonella typhymurium *and* Staphylococcus aureus *in a food model system. Basti-Akhondzadeh et al. (2007) ▶, reported that ZMEO had inhibitory effects on *Salmonella typhimurium *and* Staphylococcus aureus* in brain heart infusion broth.

Cinnamon belongs to Lauraceae family and many species of cinnamon produce volatile oil on distillation. The most important cinnamon oils in world trade are those from *C. zeylanicum*, *C. cassia,* and *C. camphora *(Jayaprakasha et al., 2007 ▶). Cinnamon leaf and bark are used as spices and in the production of essential oils. Cinnamon provides a variety of oils with different aroma characteristics and composition to the flavor industry. Total phenolic contents, chelating capacities, and radical-scavenging properties of black peppercorn, nutmeg, rosehip, cinnamon, and oregano leaf were evaluated and concluded that cinnamon had the highest natural phenolic contents and strongest antioxidant properties among the five tested botanicals (Su et al., 2007 ▶).

The present study evaluated the utilization of ZMEO and CZEO at different levels as a natural antioxidant and antifungal sources, compared with BHA and control samples in the sponge cakes and determined effect of ZMEO and CZEO on organoleptic properties and acceptability of the produced functional cakes.

## Materials and Methods

**Reagents**

Refined soy bean oil without antioxidant was provided from Pars Ghoo Factory (Tehran, Iran). Flour without preservatives, salt, and baking powder was provided from Golha Factory (Tehran, Iran). Sugar and egg were purchased from local stores. Ethanol, sodium thiosulphate, sodium hydroxide, thiobarbituric acid, BHA, peptone water, culture medium DG18, and chemical solvents were purchased from Merck Chemical Co. (Darmstadt, Germany) and DPPH˚ provided from Fluka (Germany). 

**Extraction of essential oil**

Air-dried aerial parts of *Z. multiflora *Boiss. (herbarium no. 21-13) and barks of *Cinnamon zeylanicum* were used to steam distillation by using Clevenger-type apparatus. The essential oils of the air-dried material were analysed using GC/MS.

**Preparation of the cakes**

Sponge cakes were prepared by creaming soy bean oil (without antioxidant) in 1046 g, sugar (942 g), salt (5.3 g) for 12 minutes at medium speed until light and fluffy in a Berjaya mixer (Malaysia). The ZMEO and CZEO added to the oil at three levels (500, 1000, and 1500 ppm). Whole eggs (880 g) were then slowly added at slow speed, and this was followed by mixing for 1 minute. Flour (1046 g) and baking powder (18.8 g) were added at low speed for 1 minute. The batters were baked at 180 ˚C for 20 minutes. The cakes were cooled, packed in polypropylene films, and stored at ambient conditions for 60 days. Control sample was prepared without any antioxidant and preservative. The other samples were prepared by adding BHA (100 and 200 ppm) and different levels of ZMEO and CZEO (500, 1000, and 1500 ppm).

**Antioxidant activity assay**

Peroxide value (PV), thiobarbituric acid value (TBA), and free fatty acid (FFA) content were taken as the parameters for the assesment of ZMEO and CZEO on oxidation of the cakes during 60 days of storage at 25 ˚C. PV, FFA, and TBA values were determined according to the previously reported methods (AOCS,1989 ▶; Bhanger et al., 2008 ▶; AOCS, 2006 ▶), respectively. The above analysis were carried out in three replicates and all data were averaged.

**Antifungal activity assay**

Antifungal activities of ZMEO and CZEO in the produced cakes were determined according to the ISO method (ISO21527-1, 2008 ▶). Fungal growth was determined after 1, 10, 30, 45, and 60 days of storage at 25 ˚C and reported as the ratio of colony number of samples containing ZMEO and CZEO to control cake. All experiments were carried out in triplicates.

**Organoleptic properties**

Sensory evalauation of the samples was performed using a 5-point Headonic scale. Twenty trained panelists were selected and six different codes were given to the them. Organoleptic scores for different attributes such as color, flavor, texture, and overal quality were obtained.

**Statistical analysis**

Experimental and organoleptic data were analyzed for variance (ANOVA) and significant differences (LSD test) by using the SPSS software. Data were expressed as means of triplicate analyses±SD. Differences were significant at p<0.05.

## Results

**Antioxidant activity of ZMEO and CZEO in the cake**

Changes occuring in the PV of cakes during storage are given in Table 1. PV is an index for measuring of oxidation of oil, fat and fatty foodstuffs. Peroxide produced during early stages of oxidation may change to stable materials following final step of free radical chain mechanism. TBA value of samples estimates the extent of secondary oxidation products during storage period (Table 2). 

**Table 1:** Peroxide value (g equiv. of O_2_/100 g) of the samples during 60 days of storage at 25 ˚C

	**Day**						
**Sample**	**1**	**5**	**8**	**15**	**30**	**45**	**60**
**Z**_500_	0.34±0.01^e^	0.48±0.02^g^	0.74±0.03^h^	1.17±0.02^f^	2.19±0.03^f^	2.66±0.02^f^	2.77±0.03^g^
**Z**_1000_	0.31±0.01^f^	1.04±0.04^a^	1.18±0.02^c^	1.68±0.02^c^	2.84±0.01^c^	3.11±0.02^d^	3.84±0.03^e^
**Z**_1500_	0.69±0.02^b^	0.79±0.02^d^	0.95±0.02^f^	2.24±0.03^b^	3.19±0.03^b^	4.23±0.03^b^	4.9±0.03^c^
**C**_500_	0.34±0.02^e^	0.67±0.02^fe^	0.87±0.04^g^	1.23±0.01^f^	2.33±0.01^e^	2.64±0.01^f^	3.01±0.03^f^
**C**_1000_	0.22±0.02^g^	0.68±0.02^e^	1.11±0.02^d^	1.22±0.01^f^	2.46±0.01^d^	3.79±0.02^c^	3.96±0.02^d^
**C**_1500_	0.44±0.05^d^	0.62±0.01^f^	1.14±0.01^cd^	1.57±0.01^d^	1.99±0.02^g^	2.25±0.01^h^	2.25±0.01^h^
**BHA**_100_	0.71±0.02^b^	0.99±0.03^b^	1.33±0.03^b^	1.48±0.03^e^	1.95±0.04^g^	2.97±0.03^e^	6.12±0.03^b^
**BHA**_200_	0.59±0.02^c^	0.89±0.03^c^	1.06±0.01^e^	1.19±0.02^f^	1.64±0.02^h^	2.45±0.05^g^	4.85±0.05^c^
**Control**	0.83±0.01^a^	1.10±0.05^a^	2.13±0.04^a^	2.70±0.10^a^	3.49±0.05^a^	5.5±0.05^a^	9.37±0.05^a^

Z_500_, Z_1000 _, Z_1500_, C_500_, C_1000_, and C_1500_: ZMEO and CZEO at concentrations of 500, 1000, and 1500 ppm in the cake, respectively. BHA_100_ and BHA_200_: BHA at concentrations of 100 and 200 ppm. Control: cakes without any antioxidant and preservative

**Table 2:** Thiobarbituric acid value (mg MDA/kg oil) of the samples during 60 days of storage at 25 ˚C

	**Day**						
**Sample**	**1**	**5**	**8**	**15**	**30**	**45**	**60**
**Z**_500_	0.05±0.03^c^	0.06±0.03^e^	0.1±0.01^cd^	0.13±0.02^ef^	0.21±0.03^d^	0.26±0.03^bc^	0.28±0.05^d^
**Z**_1000_	0.04±0.01^cd^	0.07±0.01^cd^	0.12±0.03^bc^	0.15±0.03^cde^	0.23±0.05^bc^	0.27±0.04^b^	0.3±0.01^d^
**Z**_1500_	0.05±0.02^c^	0.08±0.01^cd^	0.1±0.02^cd^	0.23±0.04^a^	0.28±0.1^a^	0.31±0.06^a^	0.34±0.04^b^
**C**_500_	0.05±0.01^c^	0.06±0.02^e^	0.08±0.02^d^	0.11±0.02^f^	0.16±0.02^f^	0.20±0.01^d^	0.24±0.01^e^
**C**_1000_	0.03±0.01^d^	0.13±0.02^a^	0.13±0.03^b^	0.16±0.02^cd^	0.23±0.03^bc^	0.28±0.01^b^	0.3±0.02^cd^
**C**_1500_	0.03±0.01^d^	0.06±0.01^de^	0.1±0.01^cd^	0.14±0.02^def^	0.15±0.01^f^	0.19±0.02^d^	0.22±0.01^f^
**BHA**_100_	0.08±0.03^b^	0.1±0.03^b^	0.13±0.03^b^	0.17±0.02^c^	0.23±0.03^c^	0.28±0.05^b^	0.32±0.05^bc^
**BHA**_200_	0.07±0.02^b^	0.08±0.02^bc^	0.11±0.01^bc^	0.15±0.03^cd^	0.19±0.02^e^	0.25±0.03^c^	0.28±0.04^d^
**Control**	0.09±0.01^a^	0.13±0.04^a^	0.16±0.05^a^	0.2±0.06^b^	0.25±0.04^b^	0.31±0.05^a^	0.39±0.07^a^

Z_500_, Z_1000 _, Z_1500_, C_500_, C_1000_, and C_1500_: ZMEO and CZEO at concentrations of 500, 1000, and 1500 ppm in the cake, respectively. BHA_100_ and BHA_200_: BHA at concentrations of 100 and 200 ppm. Control: the cakes without any antioxidant and preservative

Generally, PV and TBA value of all samples which contain BHA (100 and 200 ppm) and ZMEO and CZEO (500, 1000, and 1500 ppm) were lower (p<0.05) than control samples during 60 days of storage.

An increasing trend of PV and TBA value as a function of storage period, was observed for all of the samples. However, the cakes containing BHA, CZEO, and ZMEO had lower (p<0.05) PV and TBA value than the control samples at corresponding storage period. At the end of storage period (60 days), PV of control samples was 9.37±0.05 g equiv. of O_2_/100 g oil, while the PV of the other cake samples, ranged from 2.25±0.01 to 6.12±0.03 g equiv. of O_2_/100 g. ZMEO at three levels (500, 1000, and 1500 ppm) showed lower (p<0.05) PV than control samples and BHA_100_ at 60^th^ day. ZMEO at 500 and 1000 ppm had lower (p<0.05) PV than BHA_200_, but ZMEO at 1500 ppm showed no significant difference compared with BHA_200_. CZEO at three concenterations had lower (p<0.05) PV than control and BHA (100 and 200 ppm). Among all samples, CZEO at 1500 ppm had the lowest PV during 60 days of storage. TBA value of control samples was 0.39±0.07 mg MDA/kg oil, while the values of other samples ranged between 0.22±0.01 to 0.34±0.04 mg MDA/kg oil and CZEO at three levels and ZMEO (500 and 1000 ppm) showed lower (p<0.05) TBA value than BHA_100_, while ZMEO at 1500 ppm showed no significant difference compared with BHA_100_. ZMEO (500 and 1000 ppm) showed no significant difference compared with BHA_200_ and ZMEO at 1500 ppm had more (p<0.05) TBA value than BHA_200_. CZEO at 500 and 1500 ppm had lower TBA value than BHA_200_. Among all samples, CZEO at 1500 ppm had the lowest TBA value during 60 days of storage.

Changes in the FFA values of cake samples are given in Table 3. An increase in FFA value was observed in all the samples during 60 days of storage. The increase was higher (p<0.05) in the control cakes than the cake samples containing ZMEO, CZEO, and BHA, while not significant diffrence was observed among three levels of ZMEO (500, 1000, and 1500 ppm) during 60 days of storage. This result can be seen in three levels of CZEO, too. CZEO at all levels had the lowest FFA content.

**Table 3:** FFA content (% of oleic acid) of the cakes during 60 days of storage at 25 ˚C.

	**Day**						
**Sample**	**1**	**5**	**8**	**15**	**30**	**45**	**60**
**Z**_500_	0.04±0.01^d^	0.10±.02^b^	0.12±0.03^d^	0.18±0.03^bc^	0.19±0.03^e^	0.23±0.03^e^	0.33±0.06^d^
**Z**_1000_	0.08±0.03^b^	0.11±0.03^a^	0.18±0.03^a^	0.21±0.04^ab^	0.23±0.04^d^	0.27±0.05^d^	0.36±0.05^d^
**Z**_1500_	0.04±0.01^d^	0.10±0.01^b^	0.16±0.04^b^	0.16±0.01^c^	0.21±0.03^de^	0.27±0.06^d^	0.32±0.04^de^
**C**_500_	0.07±0.02^bc^	0.10±0.01^b^	0.12±0.02^d^	0.17±0.02^bc^	0.17±0.03^f^	0.22±0.03^e^	0.27±0.01^f^
**C**_1000_	0.06±0.01^c^	0.12±0.02^a^	0.15±0.01^bc^	0.18±0.04^bc^	0.21±0.05^de^	0.23±0.04^e^	0.26±0.01^f^
**C**_1500_	0.07±0.02^bc^	0.13±0.01^a^	0.17±0.03^ab^	0.19±0.02^bc^	0.22±0.04^de^	0.23±0.02^e^	0.28±0.03^ef^
**BHA**_100_	0.07±0.01^bc^	0.11±0.02^ab^	0.13±0.02^d^	0.18±0.03^bc^	0.87±0.05^c^	3.31±0.4^c^	8.02±0.5^b^
**BHA**_200_	0.10±0.01^a^	0.11±0.03^ab^	0.12±0.01^d^	0.19±0.02^bc^	1.09±0.04^a^	4.13±0.2^b^	7.08±0.6^c^
**Control**	0.10±0.02^a^	0.11±0.03^ab^	0.14±0.03^cd^	0.23±0.04^a^	0.93±0.07^b^	5.86±0.6^a^	9.32±0.7^a^

Z_500_, Z_1000 _, Z_1500_, C_500_, C_1000_, and C_1500_: ZMEO and CZEO at concentrations of 500, 1000, and 1500 ppm in the cake, respectively. BHA_100_ and BHA_200_: BHA at concentrations of 100 and 200 ppm. Control: the cakes without any antioxidant and preservative

**Antifungal activity of CZEO and ZMEO in the cakes**

**Figure 1:**
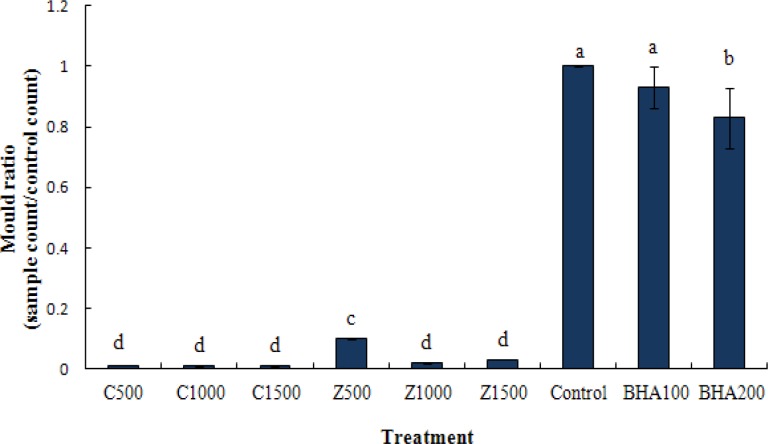
Mould ratio of sample to control after 60 days of storage at 25 ˚C. Columns with the same letters are not significantly differrent (p<0.05).

The cakes containing CZEO and ZMEO showed lower (p<0.05) percent of fungal contamination compared with the control samples during storge. On 60^th^ day, no significant difference was observed between ZMEO at 1000 and 1500 ppm and three levels of CZEO. CZEO and ZMEO at all concenterations decreased percentage of fungal growth compared with BHA (100 and 200 ppm) and control samples (Figure 1). 

**Organoleptic properties of the cakes**

**Figure 2:**
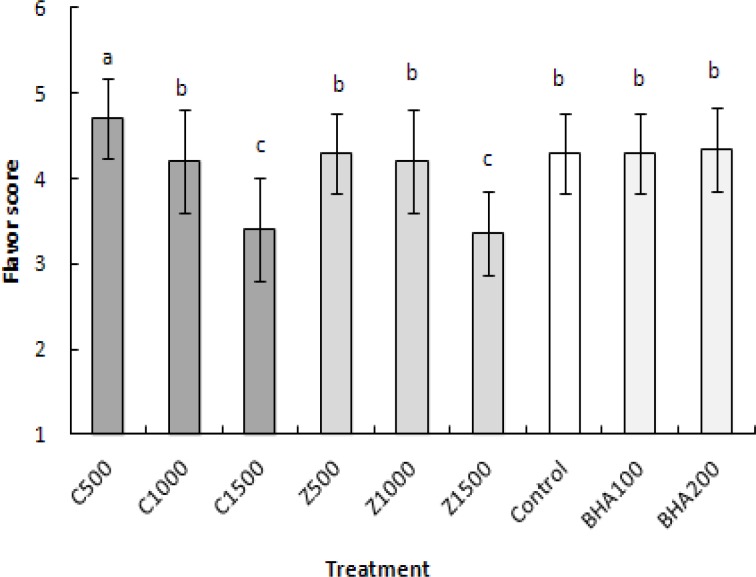
The mean values of flavor scores of the samples. Columns with the same letters are not significantly differrent (p<0.05).

It was observed that all of samples had scored higher than 4, and no significant difference was observed between them in color and texture properties. In control samples, the mean of flavor property did not differ from BHA (100 and 200 ppm), ZMEO (500 and 1000 ppm), and CZEO at 1000 ppm. ZMEO and CZEO at 1500 ppm had the lowest (p<0.05) score (Figure 2) among all samples in flavor property. About the overal quality, no significant difference was observed among ZMEO (500 ppm), BHA (100 and 200 ppm) and control samples. CZEO at 500 and 1000 ppm had the highest score of overal quality. ZMEO (1000 and 1500 ppm) showed lower (p<0.05) overal quality than other samples. Finally, results showed that total acceptance of the cakes containing CZEO and ZMEO (500 ppm) had no significant difference with control and samples containing BHA (100 and 200 ppm), while the sample containing ZMEO and CZEO (1500 ppm) showed the lowest score (p<0.05) among all samples.

## Discussion

GC/MS analysis of the ZMEO led to the identification and quantification of 28 components which accounted for 98.92% of the total oil. Carvacrol (26.08%), p-cymene (20.34%), thymol (17.23%), and linalool (10.09%) were the most abundant components and comprised 73.74% of the oil (Shahsavari et al., 2008 ▶). Moosavy et al. (2008) ▶ observed that the main components of ZMEO were carvacrol (71.1), γ-terpinene (7.34%), α-pinene (4.26%), and eucalyptol (3.37%). In 1997, Shaffiee and Javidnia (1997) ▶ found that the major components of ZMEO prepared from Yazd Province of Iran were carvacrol (61.29%) and thymol (25.18%). Sharififar et al. (2007) ▶ observed that thymol (37.59%), carvacrol (33.6%), ρ-cymene (7.72%), γ-terpinene (3.88%), and β-caryophyllene (2.06%) were the main components of ZMEO. GC/MS analysis of the CZEO identified 53 components which accounted for 94.42% of the total oil. The most components of CZEO were cinnamaldehyde (47.78%), methyl eugenol (6.75%), δ-cadinene (4.68%), and γ-cadinene (3.13%). These results were according to research of Jayaprakasha et al. (1997) ▶. The composition of the essential oil of plants can change extensively depending upon the geographicaly conditions, variety, age of the plant, and the method of drying and extraction of the oil (Bagamboula et al., 2004 ▶; Valero and Salmeron, 2003 ▶).

ZMEO and CZEO were used in the present study as functional sources. The samples were analyzed for antioxidant and antifungal properties. All levels of ZMEO and CZEO exhibited a good antioxidant and antifungal properties compared with control sample. The differences in TBA value, PV, and FFA between treated and control cakes were significant (p<0.05) in most of the cases from the first until 60^th^ day of storage. The majority of the antioxidant activity may be from compounds such as flavonoids, isoflavones, flavones, anthocyanin, catechin, and other phenolic compounds (Jayaprakasha et al., 2007 ▶). Antioxidant properties of thymol, carvacrol, and γ-terpinene were reported by Ruberto and Baratta (2000) ▶. Therefore, antioxidant activity of ZMEO can be described by the high contents of these components. It seems that the degree of antiradical property of the cinnamon essential oil may be attributed to the extent of its phenolic content (Jayaprakasha et al., 2007 ▶). Eugenol, cinnamaldehyde, cinnamic acid, and cineol were responsible for the antiradical activity of cinnamon (Mathew and Abraham, 2006 ▶). Content of FFA is described as measure of food rancidity. FFA is formed result of hydrolysis of triglycerides and may be increased by reaction between oil and moisture (Frega et al., 1999 ▶) and fungal growth may be due to increase FFA.

Common molds which exist in cakes and bakery products are *Rhizopus stolonifer, Penicilliun expansum, Penicillium stoloniferum, Aspergillus niger, Monilia sitophila, *and species of *Mucor* and *Geotrichum*. Among these, *Penicillium expansum, Penicilium stoloniferum, *and *Mucor* can produce mycotoxin (Lean and Mohamed, 1999 ▶). According to the results of chemical composition of ZMEO and CZEO, their antifungal nature apparently belonged to high phenolic contents of ZMEO and CZEO, particularly carvacrol, thymol, cinnamaldehyde, and eugenol. These findings are in agreement with other reports (Cosentino et al., 1999 ▶). Concentrations as low as 25 ppm of cinnamon essential oil inhibited germination of *Mucor* and *Aspergillus* spores after 6–8 h (Lopez et al., 2007 ▶).

ZMEO containing 26.08% carvacrol which is lipophilic component, affected the cell membrane and caused important morphological damages and disrupted membranes and the contents of the cells appeared to be depleted and amorphous. These were similar to the findings of Yamazaki et al. (2004) ▶ who found significant effect of carvacrol and thymol on *Listeria monocytogenes*. Carvacrol as the main constituent of ZMEO is a biocidal compound which causes bacterial memberane changes that lead to leakage of interacellular ATP and potassium ions and finally, death of cells (Bounatirou et al., 2007 ▶). Other components such as γ-terpinene, have been shown good antimicrobial activity probably due to synergisitic or antagonistic properties (Vardar-Unlu et al., 2003 ▶). Cinnamon effectively inhibits growth of bacteria, yeasts and molds. Its essential oil contains cinnamaldehyde and eugenol as major antimicrobial compounds (Yuste and Fung, 2003 ▶).

According to the results of studying antioxidant, antifungal, and organoleptic properties, it is indicated that CZEO and ZMEO have been effective on preservation of the cakes and they can be used for producing functional foods. It was observed that CZEO and ZMEO were efficient antioxidant and antifungal compounds during 60 days of storage. Therefore, it can be assumed that CZEO and ZMEO were stable during baking and ambient conditions used in this study.

Food industries need antioxidants that resist at high temperature used during baking, frying, and drying. Food producers need to find possible replacements to synthetic preservatives and antioxidants because of disadvantages of synthetic compounds on human body. 

This study was done to use CZEO and ZMEO as preservatives in the sponge cakes. Results of antioxidant activity, antifungal, and organoleptic properties showed that CZEO and ZMEO at concentration of 500 ppm could be used instead of BHA (100 and 200 ppm). The cakes containing CZEO and ZMEO might have considerably nutritive and functional advantages when compared with BHA and control samples. Consumption of this kind of products can help us in preventation and improvement of health disorders caused by oxidation, such as aging, atherosclerosis, and carcinogenesis. In addition, since antioxidant and antifungal properties of the different essential oils couldn’t be predicted based on their chemical composition, evaluation of the antifungal and antioxidant properties in experimental assays should always be included in their characterization.

## References

[B1] Ahmadi F, Sadeghi S, Modaressi M, Abiri R, Mikaeli A (2010). Chemical composition, invitro anti-microbial, antifungal and antioxidant activities of essential oil and methanolic extract of Himenocraterlongiflorus Benth., of Iran. Food Chem Toxicol.

[B2] Ali MS, Saleem M, Ali Z, Ahmad VU (2000). Chemistry of Zataria multiflora (Lamiaceae). Phytochemistry.

[B3] AOCS, D. Firestone (1989). Official Methods and Recommend Practices of American Oil Chemist Society.

[B4] AOCS (2006). 2-Thiobarbituric acid value direct method. Cd.

[B5] Badei AZ, Hemeda HH, Hafez SA, Hassanen NH (2000). Effect of baking and storage on the essential oil components of anise and cumin biscuits. Egypt J Agric Res.

[B6] Bagamboula CF, Uyttendaele M, Debevere J (2004). Inhibitory effect of thyme and basil essential oils, carvacrol, thymol, estragol, linalool and p-cymene towards Shigellasonnei and S. flexneri. Food Microbiol.

[B7] Bajaj S, Urooj A (2006). Effect of incorporation of mint on texture, colour and sensory parameters of biscuits. Int J Food Prop.

[B8] Basti-AkhondzadehA, Misaghi A, Khaschabi D (2007). Growth response and modelling of the effects of Zataria multiflora Boiss. essential oil, pH and temperature on Salmonella typhimurium and Staphylococcus aureus. Lebens.-Wiss. U-Technol.

[B9] Bhanger MI, Iqbal S, Anwar F, Imran M, Akhtar M, Zia-ul-Haq M (2008). Antioxidant potential of rice bran extracts and its effects on stabilization of cookies under embient storage. Int J Food Sci Technol.

[B10] Bounatirou S, Smiti S, Miguel M G, Faleiro L, Rejeb M N, Neffati M, Costa MM, Pedro LG (2007). Chemical composition, antioxidant and antibacterial activities of the essential oils isolated from Tunisian Thymus capitatus Hoff. Food Chem.

[B11] Cosentino S, Tuberoso CIG, Pisano B, Satta M, Mascia V, Arzedi E (1999). In-vitro antimicrobial activity and chemical composition of Sardinian thymus essential oils. Lett Appl Microbiol.

[B12] Fazel M, Sahari MA, Barzegar M (2009). Comparison of tea and sesame seed oils as two natural antioxidants in a fish oil model system by radical scavenging activity. Int J Food Sci Nutr.

[B13] Frega N, Mozzen M, Lercker G (1999). Effects of free fatty acids on oxidative stability of vegetable oils. J Am Oil Chem Soc.

[B14] Hosseinzadeh H, Ramezani M, Salmani G (2000). Antiinociceptive, anti-inflammatory and acute toxicity effects of Zataria multiflora Boiss extracts in mice and rats. J Ethnopharmacol.

[B15] ISO 2527-1 (2008). Microbiology of food and animal feeding Stuffs-Horizontal method for the enumeration of yeasts and moulds- part1:Colony count technique in products with water activity greater than 0.95.

[B16] Jafari Z, Boskabady MH, Pouraboli I, Babazade B (2011). Zataria multiflora Boiss inhibits muscarinic receptors of incubated tracheal smooth muscle with propranolol. Avicenna J Phytomed.

[B17] Jayaprakasha GK, Negi PS, Jena BS, Roa L (2007). Antioxidant and antimutagenic activities of Cinammon zeylanicum fruit extracts. J Food Compos Anal.

[B18] Jayaprakasha GK, Singh RP, Sakariah KK (1997). Limonoids from Citrus reticulate and their mould inhibiting activity in mosquito Culex quinquefasciatus larvae. Phytochem.

[B19] Lean LP, Mohamed S (1999). Antioxidative and antimycotics of turmeric, lemon-grass, betel leaves, clove, black pepper leaves and Garcinia atriviridis on butter cakes. J Sci Food Agric.

[B20] Lopez A, Valdiviso JB, Palou E, Sanmartin F (2007). Aspergilus flavus growth response to cinnamon extracts and sodium benzoate mixtures. Food Control.

[B21] Mathew S, Abraham TE (2006). In vitro antioxidant activity and scavenging effects of Cinnamomum verum leaf extract assayed by different methodologies. Food Chem Toxicol.

[B22] Moosavy MH, Akhondzadeh Basti A, Misaghi A, Zahraei Salehi T, Abbasifar R, Ebrahimzadeh Mousavi HA, Emami Razavi N, Gandomi H, Noori N (2008). Effect of Zataria multiflora Boiss. essential oil and nisin on Salmonella typhimurium and Staphylococcus aureus in food model system and on the bacteria cell membranes. Food Res Int.

[B23] Moure A, Cruz JM, Franco D, Dominguez JM, Sineiro J, Dominguez H (2001). Natural antioxidants from residual sources. Food Chem.

[B24] Rajaei A, Barzegar M, Mohabati Mobarez A, Sahari M A, Hamidi Esfahani Z (2010). Antioxidant, antimicrobial and antimutagenicity activities of pistachio (Pistachia vera) green hull extract. Food Chem Toxicol.

[B25] Reddy V, Urooj A, Kumar A (2005). Evaluation of antioxidant activity of some plant extracts and their application in biscuits. Food Chem.

[B26] Ruberto G, Baratta MMT (2000). Antioxidant activity of selected essential oil components in two lipid model systems. Food Chem.

[B27] Shaffiee A, Javidnia K (1997). Composition of essential oil of Zataria multiflora. Planta Med.

[B28] Shahsavari N, Barzegar M, Sahari MA, Badi HA (2008). Investigation on the antioxidant activity of essential oil of Zataria multiflora Boiss. in soy bean oil. J Med Plants.

[B29] Sharififar F, Moshafi MH, Mansouri SH, Khodashenas M, Khoshnoodi M (2007). In vitro evaluation of antibacterial and antioxidant activities of the essential oil and methanol extract of Zataria multiflora Boiss. Food Control.

[B30] Su L, Yin J, Charles D, Zhua K, Moore J, Yu L (2007). Total phenolic contents, chelating capacities, and radical-scavenging properties of black peppercorn, nutmeg, rosehip, cinnamon and oregano leaf. Food Chem.

[B31] Valero M, Salmeron MC (2003). Antibacterial activity of 11essential oils against Bacillus cereus in tyndallized carrot broth. Int J Food Microbiol.

[B32] Vardar-Unlu G, Candan F, Sokmen A, Daferera D, Polissiou M, Sokmen M (2003). Antibacterial and antioxidant activity of the essential oil and methanol extracts of Thymus pectinatus Fisch Et Mey var. pectinatus (Lamiaceae).. J Agric Food Chem.

[B33] Whysner J, Wang CX, Zang E, Iatropoulos MJ, Williams GM (1994). Dose response of promotion of butylated hydroxyanisole in chemically initiated tumors of the rat fore stomach. Food Chem.

[B34] Yamazaki K, Yamamoto T, Kawai Y, Inoue N (2004). Enhancement of antilisterial activity of essential oil constituents by nisin and diglycerol fatty acid ester. Food Microbiol.

[B35] Yuste J, Fung DYC (2003). Evaluation of Salmonella typhimurium, Yersinia enterocolitica and Staphylococcus aureus counts in apple juice with cinnamon,by conventional media and thin agar layer method. Food Microbiol.

